# Genome-wide analysis of DNA methylation and gene expression patterns in purified, uncultured human liver cells and activated hepatic stellate cells

**DOI:** 10.18632/oncotarget.4925

**Published:** 2015-08-28

**Authors:** Adil El Taghdouini, Anita L. Sørensen, Andrew H. Reiner, Mar Coll, Stefaan Verhulst, Inge Mannaerts, Cristina I. Øie, Bård Smedsrød, Mustapha Najimi, Etienne Sokal, Aernout Luttun, Pau Sancho-Bru, Philippe Collas, Leo A. van Grunsven

**Affiliations:** ^1^ Liver Cell Biology Lab, Vrije Universiteit Brussel (VUB), Brussels, Belgium; ^2^ Department of Molecular medicine, Institute of Basic Medical Sciences, Faculty of Medicine, University of Oslo, Oslo, Norway; ^3^ Institut d’Investigacions Biomèdiques August Pi i Sunyer (IDIBAPS), Barcelona, Spain; ^4^ Department of Medical Biology, Vascular Biology Research Group, UiT, The Arctic University of Norway, Tromsø, Norway; ^5^ Université Catholique de Louvain, Institut de Recherche Expérimentale et Clinique (IREC), Laboratory of Pediatric Hepatology and Cell Therapy, Brussels, Belgium; ^6^ Department of Cardiovascular Sciences, Center for Molecular and Vascular Biology, Katholieke Universiteit Leuven (KU Leuven), Leuven, Belgium

**Keywords:** Pathology section, hepatic stellate cells, liver fibrosis, DNA methylation, epigenetics

## Abstract

**Background & Aims:**

Liver fibrogenesis – scarring of the liver that can lead to cirrhosis and liver cancer – is characterized by hepatocyte impairment, capillarization of liver sinusoidal endothelial cells (LSECs) and hepatic stellate cell (HSC) activation. To date, the molecular determinants of a healthy human liver cell phenotype remain largely uncharacterized. Here, we assess the transcriptome and the genome-wide promoter methylome specific for purified, non-cultured human hepatocytes, LSECs and HSCs, and investigate the nature of epigenetic changes accompanying transcriptional changes associated with activation of HSCs.

**Material and methods:**

Gene expression profile and promoter methylome of purified, uncultured human liver cells and culture-activated HSCs were respectively determined using Affymetrix HG-U219 genechips and by methylated DNA immunoprecipitation coupled to promoter array hybridization. Histone modification patterns were assessed at the single-gene level by chromatin immunoprecipitation and quantitative PCR.

**Results:**

We unveil a DNA-methylation-based epigenetic relationship between hepatocytes, LSECs and HSCs despite their distinct ontogeny. We show that liver cell type-specific DNA methylation targets early developmental and differentiation-associated functions. Integrative analysis of promoter methylome and transcriptome reveals partial concordance between DNA methylation and transcriptional changes associated with human HSC activation. Further, we identify concordant histone methylation and acetylation changes in the promoter and putative novel enhancer elements of genes involved in liver fibrosis.

**Conclusions:**

Our study provides the first epigenetic blueprint of three distinct freshly isolated, human hepatic cell types and of epigenetic changes elicited upon HSC activation.

## INTRODUCTION

The liver is a complex organ with strong adaptive and regenerative capacity. However, persistent injury of any etiology can lead to liver fibrosis and cirrhosis, conditions associated with high morbidity and mortality [1]. Over 80% of hepatocellular carcinoma (HCC) cases have cirrhosis and most of the remainder have moderate to advanced fibrosis, making it the primary risk factors for the development of HCC [2]. These pathologies are characterized by impairment of hepatocyte (HEP) function [3], liver sinusoidal endothelial cell (LSEC) capillarization [4] and activation of hepatic stellate cells (HSCs) and Kupffer cells (KCs) [5, 6]. Under physiological conditions, these cell types form a collaborative sinusoidal unit that ensures functional organ integrity. Accordingly, there is evidence that the different liver cell types maintain each other’s differentiated phenotype [7–10].

Using cell fate tracing techniques, activated HSCs (aHSCs) have unequivocally been identified as the major source of excessive fibrillar extracellular matrix (ECM) in the fibrotic liver [5]. This is independent of the underlying disease etiology, making HSCs the primary target for anti-fibrotic therapies across all types of liver disease. Identifying the molecular determinants defining the phenotype of the various healthy human liver cell types is an essential foundation for the recognition of disease-associated changes. Moreover, despite extensive studies of the process of HSC activation in recent years, a comprehensive characterization of human primary HSCs is still lacking. In particular, changes in gene expression and the molecular events underlying these changes remain largely uncharacterized.

Gene expression is regulated by a complex interplay between transcription factors, chromatin remodeling processes and epigenetic modifications of DNA and histones, the core components of chromatin. DNA methylation consists in the addition of a methyl group on the 5 position of a cytosine within CpG dinucleotides. DNA methylation is reversible; it contributes to the proper regulation of gene expression and gene silencing in normal cells and is often associated with long-term developmental gene silencing [11, 12]. Increasing evidence links altered DNA methylation to tissue fibrosis [13]. Widespread DNA methylation changes have been reported in fibrotic lung tissue, in experimental liver fibrosis and in advanced non-alcoholic fatty liver disease [14–17]. Involvement of DNA methylation in HSC activation has also been shown [16]. Indeed, maintenance DNA methyltransferase DNMT1 and methyl-binding protein MeCP2 have been shown to play a role in promoting the activated phenotype of HSCs by repressing genes critical in the maintenance of HSC quiescence [16, 18–20]. However, the extent to which the DNA methylome of HSCs is altered during HSC activation, and how these potential alterations correlate with changes in gene expression, remain unclear.

Post-translational modifications of histones (hPTMs) also regulate tissue- and cell type-specific gene expression patterns [21], and deregulated expression of factors regulating these modifications often lead to disease [22]. Histone modifications notably include methylation and acetylation of lysines (K) on histone H3. Trimethylation of H3K27 (H3K27me3) elicits the formation of transcriptionally repressive chromatin. In contrast, H3K27 acetylation (ac) loosens histone-DNA interactions, favoring gene expression [22]. While both H3K27me3 and H3K27ac can reciprocally mark promoters, H3K27ac is also found on active enhancers. Enhancer elements are also marked by H3K4me1 irrespective of activity, while H3K4me3 marks the transcription start site (TSS) of active and many inactive genes [23]. Combinational associations of DNA methylation and histone modifications are read by effector proteins to modulate gene expression, providing cell type and tissue identity. There is however currently no information on the nature of histone modifications associated with genes involved in human HSC activation.

In this study, we report the first comprehensive and integrative analysis of the transcriptome and genome-wide promoter DNA methylome that underpin the differentiated phenotype of HEPs, LSECs and quiescent (q)HSCs purified from healthy human liver tissue. We also provide the transcriptome, promoter DNA methylome and locus-specific changes in histone modifications upon *in vitro* activation of human primary HSCs. Our data unveil an epigenetic relationship between the different hepatic cell types despite their distinct ontogeny. They also provide the epigenetic blueprint of quiescent and activated HSCs and identify novel putative enhancer elements for key genes involved in liver fibrosis.

## RESULTS

### Cell type-specific gene expression patterns of uncultured human primary HEPs, LSECs and HSCs

**Figure 1:**
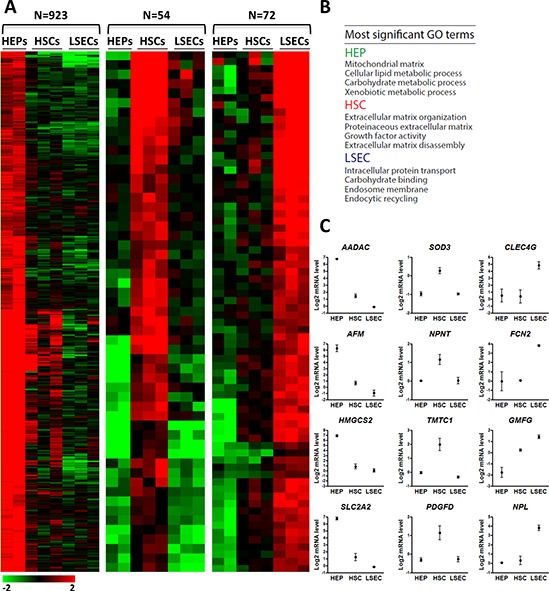
Gene expression profiling of HSCs, LSECs and HEPs identifies liver cell type selective gene expression patterns **A.** Heatmap of relative expression levels of genes classified based on expression patterns in HEPs, HSCs and LSECs. Cell type classification is based on *a* ≥ 2-fold higher expression compared to both other cell types. **B.** Most significant GO terms for each gene set shown in (A). **C.** Normalized expression level of novel indicated HEP, HSC or LSEC-specific genes.

Using a two-step collagenase perfusion technique [24] and fluorescence-activated cell sorting, we isolated HEPs, HSCs and LSECs from healthy cadaveric liver tissue and immediately processed each cell type for gene expression and promoter DNA methylation profiling (Supplementary Figure S1A–S1B). Cell purity was evaluated by differential expression of distinct liver cell type marker genes, including *CYP3A4*, *HNF4A* (HEP) [25, 26], *PDGFRB*, *VIM* (HSCs) [27–29], *CD32b* and *LYVE1* (LSEC) [30–32] (Supplementary Figure S1C). Microarray gene expression analysis reveals that 80% of all genes (*n* = 16565/20816) analyzed have similar expression levels (*P* > 0.05, ANOVA) in HSCs, LSECs and HEPs, while 20% are significantly differentially expressed in at least one of the three cell types (Supplementary Figure S1D). To identify cell type-specific genes, we focused on genes with *a* ≥ 2-fold higher expression level in one cell type relative to the two others. This reveals 923, 54 and 72 annotated genes selectively expressed in HEPs, HSCs and LSECs respectively (Figure 1A; Supplementary Table S1). Gene Ontology (GO) terms associated with these sets of genes confirm their specialized roles in metabolic processes [33], ECM homeostasis [34] and endocytosis [35], respectively (Figure 1B; Supplementary Table S2). This analysis enabled the identification of many genes with a specific expression pattern in the 3 liver cell types examined, including genes encoding for new potential cell specific surface markers (Figure 1C; Supplementary Table S1) [7–10, 36–38].

### Promoter DNA methylation marks distinct gene sets in HEPs, HSCs and LSECs

**Figure 2:**
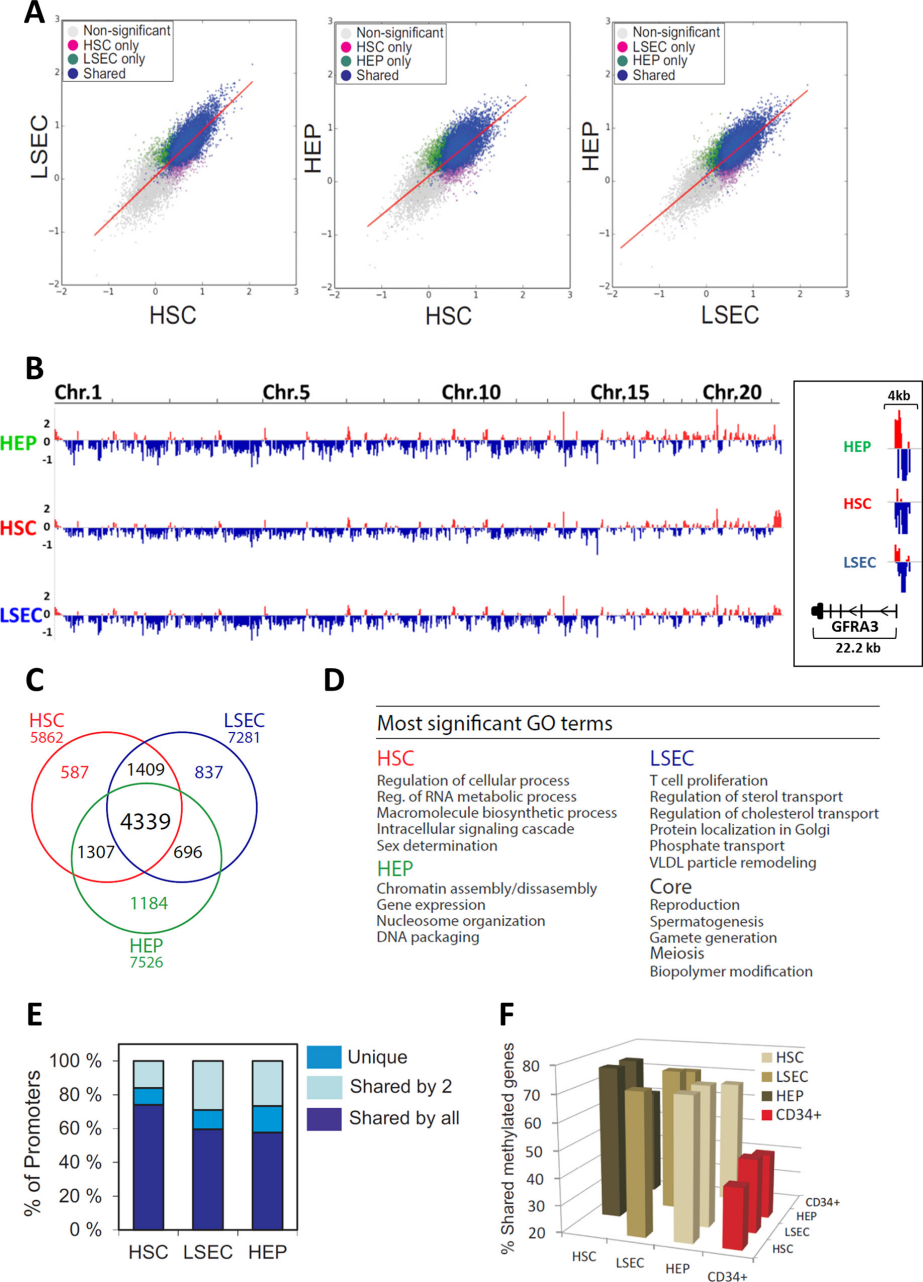
MeDIP-chip analysis of the promoter DNA-methylome of human HEPs, HSCs and LSECs **A.** Two-dimensional scatter plots of MaxTen values of methylation intensities for all promoters in HEPs, HSCs and LSECs. Genes with a promoter significantly methylated in one cell type are colored; non-significantly methylated genes are shown in gray. **B.** Browser view of promoter methylation on all chromosomes; *right*, zoom-in of *GFRA3* methylation in HEPs, HSCs and LSECs (log (MeDIP/Input) ratios). Red and blue colors point to methylation peaks and depletions, respectively. **C.** Venn diagram analysis of numbers of genes with a methylated promoter in HSCs, LSECs and HEPs. **D.** Most significant GO terms for the methylation ‘core’ and for cell type-specific methylated genes. **E.** Proportion of genes that are uniquely or commonly methylated between two or more cell types. **F.** Promoter methylation in HSCs, LSECs and HEPs relative to CD34^+^ bone marrow progenitors. Percentage of methylated genes in cell types shown on the x-axis that are also methylated in cell types shown on the y-axis.

The unexpected overall similarity of gene expression patterns detected in purified, non-cultured HEPs, HSCs and LSECs suggests an “intrinsic” identity of these cell types despite their distinct function. Gene expression patterns are largely determined by reversible epigenetic modifications. Thus, we assessed the epigenetic relationship between uncultured HEPs, HSCs and LSECs by methylated DNA immunoprecipitation coupled to promoter array hybridization (MeDIP-chip). We examined methylation profiles through 4 kilobases (kb) of genome across all human RefSeq promoters, spanning −3 to +1 kb relative to the transcription start site (TSS). Correlations of MeDIP/Input log2 ratios show high reproducibility between technical replicates (*r* > 0.95; data not shown). Pair-wise comparisons of MaxTen values of DNA methylation intensities for all promoters (see Methods; Figure 2A) and browser views of promoter methylation profiles show overlap but also differences between cell types (Figure 2B).

Combining MeDiP-chip data from two donors for each cell type (Supplementary Figure S2A), we identify with high confidence (KS test, *P* ≤ 0.01) a total of 7526 genes with a methylated promoter in HEPs, 5862 in HSCs and 7281 in LSECs, representing 30–35% of all RefSeq promoters (Figure 2C; Supplementary Figure S2A, intersects; Supplementary Table S3). We also identify a “core” of 4339 methylated genes, illustrating similarity in the promoter methylomes of these three cell types (Figure 2C). GO enrichment analysis shows that this gene core mainly pertains to early developmental and reproduction/gametogenesis-associated functions (Figure 2D), consistent with long-term repressive DNA methylation of reproduction-associated genes in the soma [39, 40]. Interestingly, ~600 to ~1200 genes emerge as uniquely methylated in either cell type (Figure 2C); these are linked to functions pertaining to chromatin assembly and gene regulation (HEP), RNA metabolism and signalling (HSC) and intracellular transport and lipid metabolism (LSEC) (Figure 2D; Supplementary Table S4). We conclude that 60 to nearly 75% of methylated genes are shared between HEPs, HSCs and LSECs, while 10–16% are uniquely methylated in either cell type (Figure 2E; Supplementary Table S5). Thus, while these cell types share a common promoter methylation pattern, they are also characterized by some epigenetic diversity.

We next evaluated the extent to which the promoter methylome of HSCs, LSECs and HEPs was similar to that of an unrelated, also uncultured, cell type, such as bone marrow-derived CD34^+^ hematopoietic progenitors, which we have previously analyzed by MeDIP-chip [40]. We find that 42–47% of promoters methylated in CD34^+^ cells are also methylated in HSCs, LSECs or HEPs (Figure 2F). Thus, at a promoter DNA methylome level, bone marrow CD34^+^ cells show a similar ‘epigenetic distance’ to any of the three liver cell types examined. Moreover, promoters methylated in HSCs, LSECs and HEPs as a whole compared to CD34^+^ cells are mainly involved in cell cycle regulation (data not shown). These results suggest that, despite functional and ontology differences between HSCs, LSECs, HEPs and CD34^+^ hematopoietic progenitors, the ‘epigenetic distance’ between the three liver cell types is shorter than between any of these cell types and CD34^+^ cells.

### Human HSC activation is associated with a profound change in gene expression and DNA methylation

**Figure 3:**
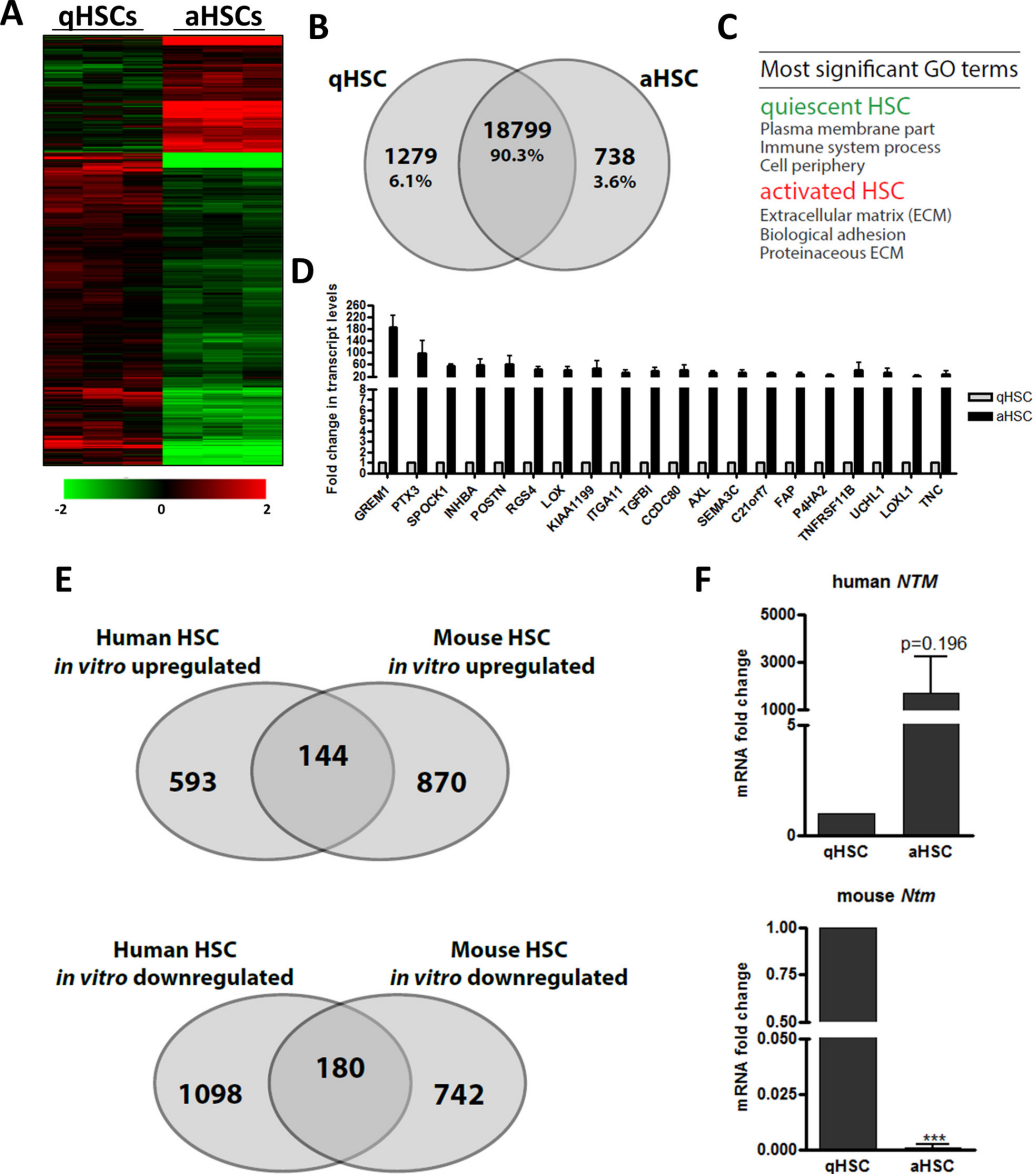
The gene expression changes elicited by *in vitro* HSC activation poorly correlate between mouse and human **A.** Heatmap of relative expression levels of genes classified based on differential expression (≥ 2-fold) between human qHSCs and culture induced aHSCs. **B.** Venn diagram analysis of numbers of genes (absolute and as percentage of total analyzed genes) shown in (A). **C.** Enriched GO terms for genes differentially expressed between qHSCs and aHSCs. **D.** Normalized, relative expression levels of the top 20 most-upregulated genes following human HSC activation *in vitro*. **E.** Venn diagram analysis illustrating the overlap of annotated genes differentially regulated following *in vitro* activation of human and mouse HSCs. Mouse data from [59]. **F.** Relative neurotrimin (*NTM*) mRNA expression levels in freshly isolated, non-cultured qHSCs and culture aHSCs from human and mouse.

We next determined changes in transcriptome and promoter DNA methylome associated with human HSC activation. To this end, we compared freshly isolated, uncultured qHSCs with culture-activated HSCs (aHSCs). We find that over 2000 genes are differentially expressed upon HSC activation, representing ~10% of the total genes examined and consisting primarily of down-regulated (6.1%) and fewer up-regulated (3.6%) genes, with the latter ones being mainly linked to the ECM (Figure 3A–3C, Supplementary Table S6). This profiling allowed the confirmation of genes previously known to be associated with HSC activation, such as *GREM1* [41], *LOX* [42] and *TNC* [43]. Importantly, this also enabled identification of novel putative human *in vitro* HSC activation-associated genes (Figure 3D, Supplementary Table S6). Surprisingly, we find limited overlap (~12–18%) between the global changes in gene expression underlying *in vitro* activation of mouse primary HSCs – by far the most common model used to study HSC biology – and human primary HSCs (Figure 3E), suggesting a different transcriptional cascade underpinning HSC activation in both species. As illustrative example, neurotrimin (*NTM*), a well-known quiescence associated gene in mouse HSCs [44], was found to be strongly upregulated during human HSC activation (Figure 3F).

**Figure 4:**
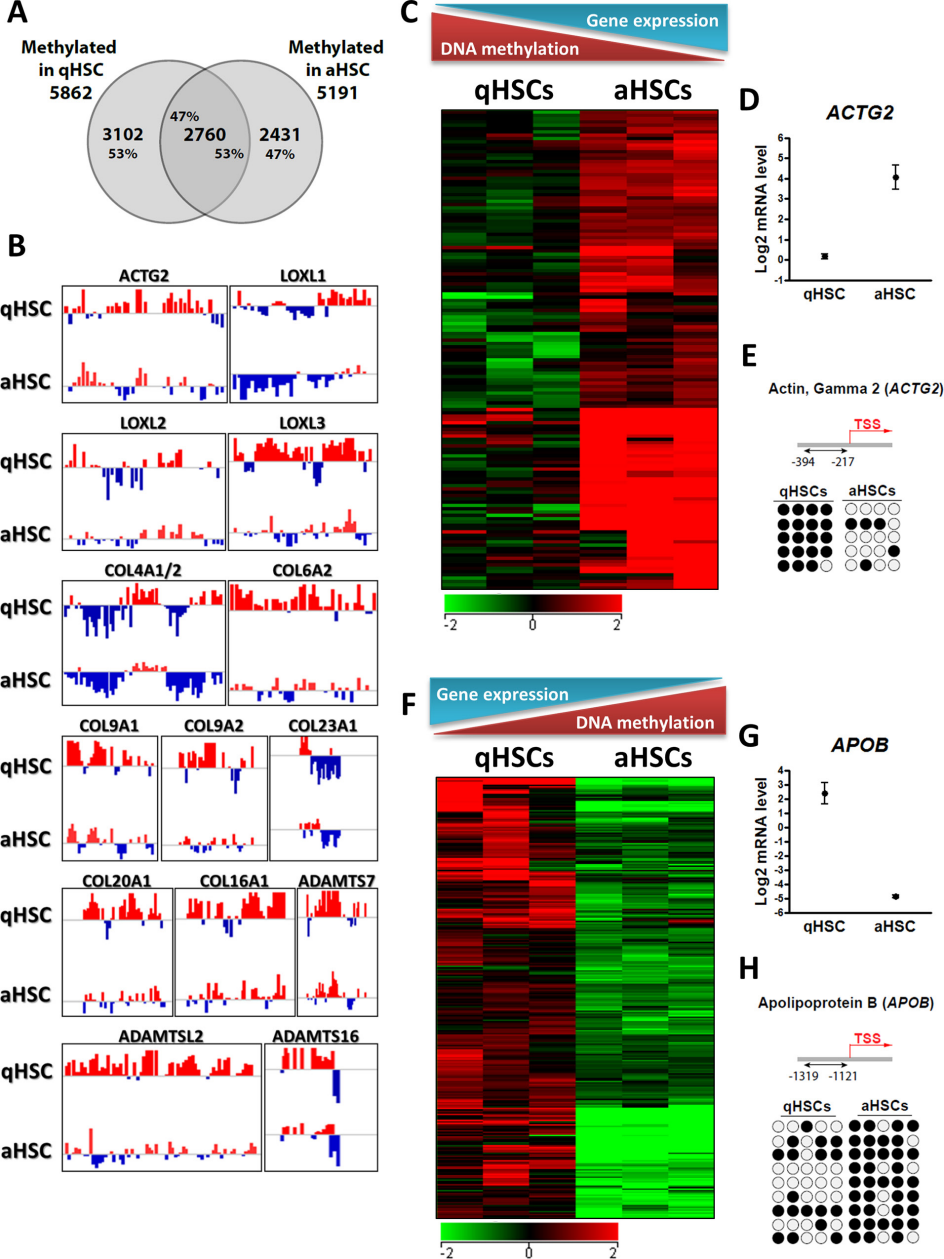
Culture-induced HSC activation reprograms promoter DNA methylation **A.** Venn diagram analysis of the number of genes with a methylated promoter in qHSCs and aHSCs. **B.** Browser views of promoter methylation profiles (log (MeDIP/Input) ratios) for indicated genes in qHSCs and aHSCs. Red and blue colors point to methylation peaks and depletions, respectively. **C.** Heatmap of genes up-regulated and hypo-methylated after HSC activation. **D.** Boxwhisker plot of *ACTG2* expression in qHSCs and aHSCs. **E.** Bisulfite sequencing analysis of CpG methylation in the *ACTG2* promoter in qHSCs and aHSCs. Four CpGs are examined (columns) in 5 sequenced clones (rows). ● methylated CpG; ○ unmethylated CpG. **F.** Heatmap of genes down-regulated and hyper-methylated after HSC activation. **G.** Boxwhisker plot of *APOB* expression in qHSCs and aHSCs. **H.** Bisulfite sequencing analysis of CpG methylation in the *APOB* promoter in qHSCs and aHSCs. Five CpGs were analyzed.

Further, we examined changes in the promoter methylome of aHSCs and find that it deviates substantially from that of qHSCs. MeDIP-chip reveals 5862 methylated genes in qHSCs and 5191 in aHSCs (Figure 4A). We identify a core overlap of only 2760 methylated genes (Figure 4A), i.e. ~50% of all methylated genes in either qHSCs or aHSCs. We note a net reduction in promoter methylation in aHSCs, with demethylation of 3102 promoters (53% of all methylated promoters before culture; Figure, 4A), including several different members of the collagen and lysyl oxidase gene families (Figure 4B), the main constituents and enzymatic stabilizers of fibrotic scar tissue [45]. *De novo* methylation of 2431 promoters was observed, representing 47% of all methylated promoters in aHSCs (Figure 4A). Demethylated genes in aHSCs are notably involved in regulation of nucleotide metabolism pertaining to cell cycle progression and signal transduction, consistent with induction of cell division upon activation and adjustments in signal transduction pathways as a result of changing conditions.

Recent work has shown that developmental transitions can be associated with differential methylation of promoter regions upstream of the TSS rather than at or downstream of the TSS [46]. We find that in qHSCs, methylation is equally distributed into upstream, downstream or at the TSS (‘TSS methylation’), with ~30% of promoters showing upstream methylation (only). In aHSCs however, upstream methylation significantly increased to 51–58% for the same two donors (L4 and L11), primarily at the expense of ‘TSS methylation’ (*P* < 0.001, Chi-Square test) (Supplementary Figure S2C). Upstream methylation is also prominent in aHSCs from two other donors (L8, L10), suggesting that this might be a common feature of HSC culture.

We find 416 genes (Supplementary Table S7) with concordant changes in DNA methylation and gene expression upon HSC activation. For these genes, transcriptional upregulation in aHSCs correlates with abrogation or reduction in promoter methylation, as shown for several pro-fibrogenic genes such as *ACTG2*, *LOXL1*, *LOXL2* and *COL4A1/2* (Figure 4B–4E). Conversely, transcriptional downregulation among these 416 genes is associated with DNA hypermethylation (e.g. *APOB*, *ADAMTS9*, *MMP15* and *CXCL9*; Figure 4F–4H). Independent bisulfite sequencing analysis of *ACTG2* and *APOB*, two genes strongly differentially expressed between human qHSCs and aHSCs, corroborates their differential methylation status detected by MeDIP-chip (Figure 4E, 4H).

To evaluate the physiological relevance of these findings, we determined whether similar DNA methylation changes would also occur in mice HSCs after a 4-week induction of fibrogenesis with carbon tetrachloride (CCl_4_) (Supplementary Figure S3A–S3C). Bisulfite sequencing analysis of *Loxl1*, *Loxl2*, *Col4a1* and *Col4a2*, which are strongly upregulated in *in vivo* activated human and mouse HSCs (Supplementary Figure S4A–S4B), shows no difference in promoter methylation between qHSCs and aHSCs (Supplementary Figure S3D). This is explained by the already unmethylated state of these genes in qHSCs isolated from control untreated mice (Supplementary Figure S3D) and suggests, rather, that methylation of at least a subset of pro-fibrogenic genes is distinct between mice and humans. Our transcriptome and methylome data collectively indicate that a significant gene set is differentially expressed with concordant DNA methylation changes upon *in vitro* activation of human qHSCs. Moreover, the difference in methylation state of specific genes between quiescent mouse and human HSCs argues for the importance of using human HSCs *in vitro* as a model system to elucidate the molecular regulation of pro-fibrotic gene activation in humans.

### HSC activation is linked to alterations in histone methylation on quiescence-associated and pro-fibrotic genes

**Figure 5:**
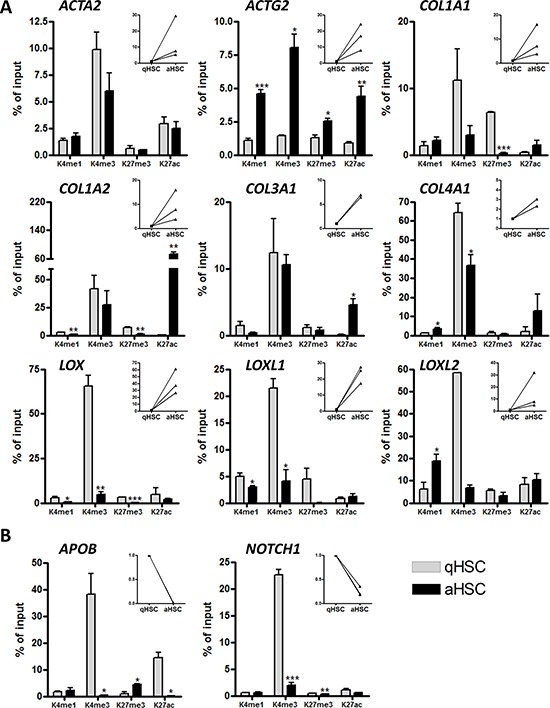
Histone H3 methylation and acetylation occupancy on promoters of aHSC- and qHSC-associated genes ChIP analysis of DNA isolated from freshly isolated human qHSCs and cultured induced aHSCs. **A.** qHSC associated genes. **B.** aHSC associated genes. The main graphs show the percentage enrichment of H3K4me1, H3K4me3, H3K27me3 and H3K27ac relative to input, in human qHSCs and aHSCs. The right insert panels show the fold increase or decrease in mRNA levels for the respective gene during human HSC activation *in vitro*.

The non-straightforward relationship between promoter DNA methylation and changes in gene expression following HSC activation prompted the query of additional chromatin marks that would account for differential gene expression. We examined by chromatin immunoprecipitation (ChIP)-qPCR the profiles of H3K4me1, H3K4me3, H3K27ac and H3K27me3 [47] on upstream regulatory regions of a set of pro-fibrogenic genes (Figure 5, Supplementary Table S8). First, we find that promoters of all genes examined are marked by H3K4me3, albeit at various levels, in either qHSCs, aHSCs, or both (Figure 5A, 5B), consistent with a marking of the TSS for transcription. The level of H3K4me3 enrichment at these sites reflects the expression level of the gene in most cases (e.g. *ACTG2* (increase), *COL1A2* (elevated) and *COL3A1* (elevated), *APOB* and *NOTCH1* (decreased) but not always (e.g. *COL1A1*, *LOX*, *LOXL1*, *LOXL2*) (Figure 5A, 5B). Because ChIP data represent an average of all cells in a population, this could reflect biological variation in the HSC culture. We find that the promoters of *COL1A1*, *COL1A2*, *COL3A1, COL4A1* and *ACTG2* are enriched in H3K27ac, coinciding with increased expression in aHSCs (see also [48]) (Figure 5A). In contrast, the quiescence genes *APOB* and *NOTCH1* show reduced H3K4me3 and/or H3K27ac after HSC activation, in line with transcriptional down-regulation in aHSCs (Figure 5B). Interestingly, we also note that the *LOX*, *LOXL1* and *COL1A2* promoters in aHSCs display reduced H3K27me3 in aHSCs, strongly suggesting a depression mechanism coinciding with transcriptional up-regulation. These results collectively indicate that gene expression changes occurring after culture-induced HSC activation are linked to, for the most part (albeit not always), concording changes in modifications of H3K4 and H3K27 on promoters.

### Identification of novel putative enhancer elements in pro-fibrotic genes

To gain further insight into histone modifications which may affect activation-induced transcriptional changes, we examined, for a subset of genes showing discordant promoter H3 methylation and expression changes (notably *COL4A1*, *LOXL1*, *LOXL2*), additional putative regulatory elements. We searched for putative enhancers for a subset of these genes, given that some enhancers cluster near genes they regulate [49]. To this end, we examined published ChIP-sequencing profiles for H3K4me1, an enhancer mark, and H3K27ac, which together with H3K4me1 characterizes active enhancers. The ChIP-seq profiles examined were previously obtained during adipogenic differentiation of primary human adipocyte progenitors [50], and were used with the rationale that HSC activation can be defined as a ‘differentiation’ from a quiescent, adipogenic-like state (qHSCs) into a fibrogenic myofibroblast-like state (aHSCs). Thus, *in vitro* HSC activation arguably displays (anti)parallels to *in vitro* adipogenesis, and anti-adipogenic regulation has been shown to underlie HSC activation [51].

**Figure 6:**
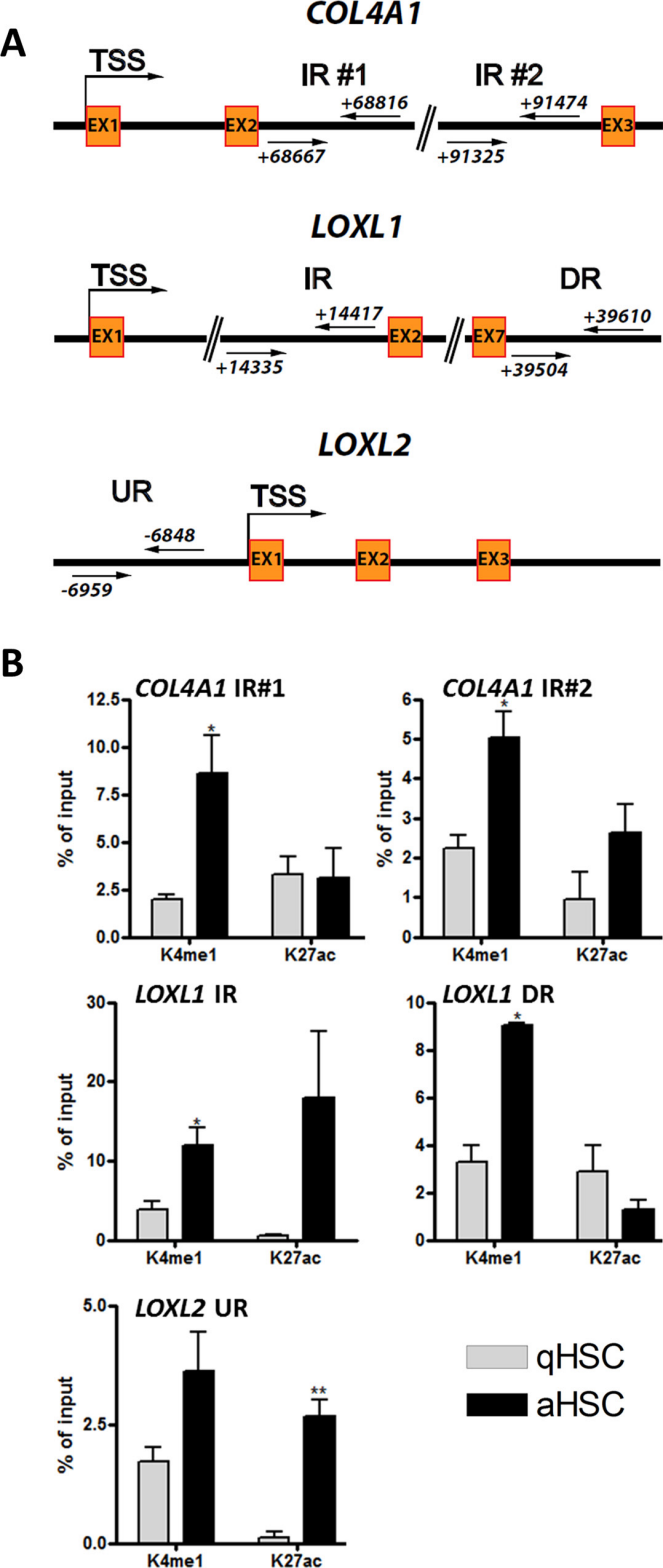
Identification of putative novel enhancer elements for the pro-fibrotic COL4A1, LOXL1 and LOXL2 genes **A.** Schematic representation of putative poised or active enhancers upstream or downstream of the *COL4A1*, *LOXL1* and *LOXL2* TSS. Two intragenic poised putative enhancer regions were identified 69 kb and 90 kb downstream of the *COL4A1* TSS. Intragenic active and downstream poised putative enhancers were identified 14 kb and 39 kb downstream of the *LOXL1* TSS respectively, and an upstream active putative enhancer was identified 6.5 kb upstream of *LOXL2* TSS. **B.** Graphs showing the percentage enrichment of H3K4me1 and H3K27ac relative to input, in human qHSCs and aHSCs, at the sites shown in (A) IR, DR, UR; Intragenic, downstream, upstream region.

Examination of chromatin marks of pro-fibrogenic genes in pre-adipocytes identifies upstream, downstream and intragenic sites at the LOXL1, LOXL2 and COL4A1 loci marked by high H3K4me1 and low H3K4me3 co-marked (or not) by H3K27ac, which together with H3K4me1 marks active enhancers (data not shown; see Figure 6A for sites examined). Interestingly, the results obtained by performing ChiPs for these sites in qHSCs and aHSCs clearly show that H3K4me1 is enriched at all sites examined after HSC activation (Figure 6B); these gains in H3K4me1 strongly suggest that (putative) enhancer elements are being ‘marked’ for transcriptional activation upon HSC activation. Additionally, for each gene, we identify an increase in H3K27ac, reflecting the use (activity) of the putative enhancer. This is particularly evident for a downstream site within the *COL4A1* gene immediately upstream of exon 3, a region upstream of exon 2 in the *LOXL1* gene, and an upstream region nearly 7 kilobases upstream of the *LOXL2* TSS (Figure 6B). Importantly, these findings point to the localization of functional enhancer elements hitherto unidentified in key pro-fibrotic genes up-regulated upon HSC activation.

## DISCUSSION

We report the first comparative transcriptome and promoter DNA methylation of freshly isolated uncultured human HEPs, HSCs and LSECs. We show that *in vitro* activation of HSCs is associated with changes in DNA methylation and histone modification on thousands of promoters including some that drive pro-fibrotic gene expression.

Cell type-specific gene sets identified by transcriptomic analysis of HEPs, HSCs and LSECs confirm their specialized roles in metabolic processes [33], ECM homeostasis [34] and endocytosis [35]. For example, we find that HSCs express *SOD3*, the major superoxide dismutase known to bind and protect vascular endothelial cells from oxygen radicals [36]. In line with a previous study identifying vascular smooth muscle cells as major source of *SOD3* in the arterial wall [37], our finding suggests a potential role for HSCs in protecting the liver-specific vasculature by suppression of various pathological processes. On the other hand, LSECs express *GMFG*, a glia maturation factor which negatively regulates lipopolysaccharide-induced TLR4 signaling in macrophages [38], suggesting a role for LSECs in the maintenance of Kupffer cell (KC) and HSC quiescence. These observations, in line with previous studies in rodent cells [7–10], suggest that liver cells promote the maintenance of each other’s phenotype.

Cell type-specific methylation is associated with processes disabled in the course of hepatic specification and development. Methylated genes in HSCs are enriched in RNA metabolism consistent with the quiescence of HSCs in healthy liver, a state associated with low RNA metabolic activity [52]. Genes methylated in LSECs are linked to lipid metabolism confirming that LSECs are the only cell type among those examined which do not have a lipid storage or processing role, unlike HSCs and HEPs [53–55]. In accordance with a developmentally repressive role of DNA methylation, these functions are likely to be repressed by DNA methylation in LSECs, epigenetically segregating them from HSCs and HEPs. Methylated genes in HEPs pertain almost exclusively to chromatin assembly functions. This may be related to extensive nuclear and chromatin remodeling taking place during hepatocyte development and maturation, often leading to binucleated cells and a substantial degree of multiploidy [56]. The methylated state of chromatin remodeling-associated genes suggests that these nuclear remodeling activities have become repressed by DNA methylation once hepatocyte maturation has occurred. Thus in the post-natal liver, promoter methylation targets early developmental functions disabled during liver organogenesis.

At the transcriptome and methylome level, LSECs and HSCs are more similar to each other than to HEPs. Nonetheless, HEPs, HSCs and LSECs seem to display a shorter ‘epigenetic distance’ between each other than to bone marrow-derived freshly isolated CD34^+^ hematopoietic progenitors [40], an unrelated cell type. A common ontogenic environment during liver development is likely to impact this epigenetic relationship [57]. This is in line with the similar transcriptome of hepatic and pancreatic stellate cells, which are thought to share a common origin [58]. This suggests that developmental origin is an important determinant of the hepatic transcriptome and methylome.

Our study demonstrates that human HSC activation is associated with extensive remodeling of the promoter methylation landscape. The link between promoter DNA methylation and gene expression is nonetheless rather promiscuous, consistent with the non-straightforward relationship between promoter methylation and expression status of the associated gene [39, 40]. Our study constitutes, to our knowledge, the first reference of transcriptome and methylome changes elicited by culture-induced activation of human HSCs. To what extent these profiles reflect fibrosis-induced HSC activation in humans [44] remains to be determined, however this is challenged by the current difficulty to isolate pure activated HSCs from fibrotic livers. Indeed, it is not until recently that ultra-pure HSC isolation procedures from fibrotic mouse livers have been published as a validated method to determine gene expression changes during HSC activation, devoid of cell culture artifacts [59, 60]. To the best of our knowledge, there is to date only one report about the isolation of aHSCs from cirrhotic human livers [61]. Of note, in the latter study both the quiescent and *in vivo* activated HSCs were *in vitro* cultured.

Recently, another DNA modification at the 5′ position of cytosine, i.e. 5-hydroxymethylcytosine (5 hmC) was discovered [62, 63]. This novel cytosine modification occurs through the oxidation of 5mC by the TET (Ten-Eleven-Translocation) family of enzymes and is believed to be a key intermediate of active DNA demethylation [64]. A limitation of the MeDIP-Chip and bisulfite sequencing approaches is that they do not allow for the discrimination of DNA methylation and hydroxymethylation. While the genomic distribution of DNA hydroxymethylation is well studied in different tissues and cell types, its role and functional significance across the genome are still unclear. While evidence suggests that like methylation, the concentration of hydroxymethylated cytosines at promoter regions negatively correlates with transcriptional activity [65], other studies have associated 5 hmC with increased transcriptional levels [66–68]. As increasing evidence suggest that both DNA modifications may have different functional outcomes, future studies to decipher the dynamics of DNA hydroxymethylation during HSC activation will provide novel insights into the role of this epigenetic modification in setting a pro-fibrogenic transcription program.

We find that around 10% of the analyzed genes are deregulated upon activation, with a majority of genes being down-regulated, and provide a list of previously known and unknown genes with a differential expression between human qHSCs and aHSCs. Although *in vitro* activation is currently the most common model of HSC activation, it incompletely replicates gene expression changes associated with *in vivo* activation, as shown in rodents [44, 59]. We show here that *in vitro* activation of human and mouse HSCs induces a different transcriptional response, emphasizing the importance of validating observations made with mouse or rat HSC cultures for relevance in human primary cells or tissue.

Importantly, our findings highlight the identification of novel putative and functional enhancer elements for pro-fibrotic genes, which epigenetically respond to culture-induced HSC activation by displaying marks of regulatory activity, namely H3K4me1 in combination with H3K27ac. This is clearly evidenced for *LOXL1*, *LOXL2* and *COL4A1*. Of note, these genes show histone modification changes on promoters that do not concord with their transcriptional activation. This suggests that their transcriptional activity is modulated by enhancer marking (and hence ‘activity’) rather than promoter marking *per se*. It is tempting to speculate that these putatively novel enhancers may play a key role in the maintenance of a pro-fibrogenic gene expression program.

## MATERIALS AND METHODS

### Patients

Gene expression and DNA methylation profiling was performed using HEPs, HSCs and LSECs isolated from cadaveric donors (Table 1). HEPs were also harvested from consenting patients undergoing hepatic resections for liver metastasis from colorectal carcinoma (CRC). The protocol and conducted experiments were approved by the ethical committees of St-Luc Hospital and faculty of Medicine of Université Catholique de Louvain, and by the Norwegian Research Ethics Committee. An agreement from the Belgian Ministry of Health was obtained for the hepatocyte and hepatic stem cell bank.

**Table 1:** Clinical characteristics of the liver cell donors

Donor number	Health status	Age	Gender	Ischemia time
**L4**	Healthy	12 years	Female	16 h 30
**L8**	Healthy	1 day	Male	4 h 40
**L10**	Healthy	7 months	Female	5 h 20
**L11**	Healthy	7 days	Male	4 h 25
**F2**	Trauma	17 years	Male	9 h
**F23**	Trauma	16 years	Female	<10 h
**B6T**	Colorectal cancer	67 years	Female	<1 h
**B5O**	Colorectal cancer	75 years	Male	<1 h

### Human liver cell isolations

Human liver cells were isolated from the left liver segment of healthy donors up to 12 hours after clamping using a two-step perfusion technique [24]. Livers were kept on ice until sequential perfusion with an EGTA and digestion enzyme solution (0.9 mg/ml collagenase *P* and 0.03 mg/ml soybean trypsin inhibitor) was performed. Hepatocytes (HEPs) were separated from the non-parenchymal liver cells by low-speed (50 g) centrifugation steps followed by two washing steps and purified by means of a Percoll gradient. Dissociated single non-parenchymal cells were suspended in a 5% FBS, 2 mM EDTA phosphate buffered saline (PBS) (10^6^cells/100μL) and incubated for 30 min at 4°C with 500 ng/10^6^ cells anti-CD32 (Abcam, Cambridge, United Kingdom) and 1 μg/10^6^ cells anti-CD45 (BD Biosciences, San Jose, CA) or corresponding isotype controls. Cells were washed twice by centrifugation and resuspended in 2 mM EDTA PBS, before FACS sorting. Enriched populations of human qHSCs were sorted through a negative selection for CD32 (Ex: 488 nm; Em: 575 nm) and CD45 (Ex: 495 nm; Em: 519 nm) expressing cells and a positive selection for ultraviolet positivity (retinyl esters are auto-fluorescent at 328 nm), using a FACS-Aria (BD Biosciences). Enriched populations of LSECs were obtained as CD32^+^CD45^−^ cells (Supplementary Figure S1A–S1B) and 7-aminoactinomycin (eBioscience, San Diego, CA) was used to discriminate viable from non-viable cells. On average, 15–20 × 10^6^ HEPs were isolated from 1 g of liver tissue and 7.3% (±7.5) HSCs and 4% (±2.1) LSECs were sorted from the total number of non-parenchymal cells. Separated populations of HEPs, qHSCs and LSECs were immediately used for total cell RNA for gene expression profiling or DNA extraction for DNA methylation profiling and chromatin immunoprecipitation (ChIP).

### *In vitro* activation of human primary HSCs

Homogeneous populations of aHSCs were obtained as described previously [69]. In brief, the qHSC-enriched population obtained after Nycodenz (Nyegaard, Oslo, Norway) gradient centrifugation were plated on plastic culture dishes (Greiner Bio-one, Monroe, NC) and allowed to activate and expand for up to 3 to 4 passages in DMEM (Lonza, Verviers, Belgium) supplemented with 10% fetal bovine serum (Biochrom GmbH, Germany) at 37°C in a humidified atmosphere with 5% CO_2_. Purified qHSC and *in vitro* aHSC populations were immediately used for total DNA extraction and bisulfite sequencing.

### Isolation of murine HSCs from healthy and 4-weeks CCl_4_-treated mice

Murine qHSC and *in vivo* aHSC populations were isolated from male BALB/c mice (Charles River Laboratories, L’Arbresle, France) (aged 12 weeks) as described [70] with some modifications. Briefly, after enzymatic perfusion of the liver and low-speed centrifugation steps to remove HEPs, HSCs were purified from the non-parenchymal cell mixture through FACS-sorting for UV-positive cells using a FACS-Aria. For *in vivo* activation of HSCs, mice underwent eight intraperitoneal injections over 4 weeks of 50 μl CCl_4_/100 g body weight in mineral oil (Sigma-Aldrich, St. Louis, MO). All procedures on animals were carried out in accordance with University’s guidelines for the care and use of laboratory animals in research and the performed experiments were approved by the ethical committee of the Vrije Universiteit Brussel in project 13-212-3.

### Gene expression profiling

Total RNA was isolated by using RNeasy MICROKit (Qiagen GmbH, Hilden, Germany) following manufacturer’s recommendations. Total RNA concentration and quality control was assessed using RNA 6000 pico kit (Agilent, Santa Clara, CA, USA). Due to the small amount of RNA obtained, RNA samples were amplified by using Ovation PicoSL System V2 (NuGene Technologies, CA, USA) and ENCORe Biotin module (NuGene technologies). RNA MinElute Reaction Cleanup Kit (Qiagen) was used to purify amplified RNA. RNA samples were labeled and hybridized by using GeneChip Hybridization Control Kit and GeneChip Hybridization, Wash and stain Kit HT (Affymetrix, Santa Clara, CA, USA), respectively. Labeled RNA samples were then hybridized to Affymetrix HG-U219 genechips (16 arrays plate) (Affymetrix) according to the manufacturer’s instructions. Data normalization and analysis was performed using GeneSpring GX12 (Agilent, Santa Clara, CA) as described previously [71]. For detection of differentially expressed genes, a *P*-value cut-off of 0.05 was used in combination with a fold-change cut-off of 2.0. Raw data are made publically available on the NCBI Gene Expression Omnibus database, with accession number GSE68000.

### RNA purification and RTq-PCR

Total cell RNA was extracted and purified using the Reliaprep RNA cell Miniprep System (Promega, Madison, WI) and converted to cDNA by reverse transcription using the Revert Aid Kit (ThermoFisher Scientific, St. Leon-Rot, Germany). Quantitative real-time polymerase chain reaction was performed using the GoTaq qPCR Master Mix with BRYTE green (Promega). A 7500 real time PCR system was used and data was analyzed using System SDS software v2.0.6 (Applied Biosystems).

### Bisulfite genomic sequencing

DNA was purified and bisulfite-converted using the EpiTect Bisulfite Kit (Qiagen). Converted DNA was amplified by PCR using primers designed using Methprimer (http://www.urogene.org/methprimer/) (Supplementary Table S8). The TSS position was determined using Ensembl (http://www.ensembl.org). PCR conditions were 95°C for 5 min and 35 cycles of 95°C 1 min, 58°C 2 min, 72°C 2 min, followed by 10 min at 72°C. PCR products were purified from an agarose gel with the GenElute Gel Extraction Kit (Sigma) and cloned into *E. coli* by TOPO TA cloning (Life Technologies) and sequenced. Methylation data are shown as methylated CpG (filled) and unmethylated CpG (empty) circles.

### Methyl-DNA immunoprecipitation and data analysis

MeDIP was performed and data were analyzed as described [40, 72]. In short, genomic DNA was purified and fragmented by sonication in a Bioruptor^™^ (Diagenode) to obtain DNA fragments of 200–500 bp. Methylated fragments (3 μg) were immunoprecipitated using 5 μg anti-5-methylcytosine antibodies (Diagenode, MAb-006.100). MeDIP and input DNA were amplified by 14 PCR cycles using the WGA2 kit (Sigma-Aldrich) and cleaned up (MinElute PCR Purification Kit; Qiagen). Input and MeDIP DNA were labeled with Cy3 and Cy5, respectively, and hybridized onto Roche-Nimblegen HG18 RefSeq Promoter arrays (No. C4226–00-01). Data analysis was done as described [40] using a one-sided Kolmogorov-Smirnov (KS) windowed test to identify probes with a significantly positive signal. Resulting score for each probe was the *P*-value from the windowed test around that probe. Using NimbleScan, methylated peak data were generated from *P*-values by querying for ≥ 2 probes with *P* ≤ 0.01.

For comparison of promoter DNA methylation between each liver cell type and bone marrow CD34^+^ cells, we focused on one donor for each liver cell type since methylation data from one CD34^+^ cell donor were originally collected [40]. Moreover, analysis was restricted to the genomic window originally examined, i.e. from −2.2 to +0.5 kb relative to TSS in order to match our earlier dataset for CD34^+^ cells (see Supplementary Figure S2B).

### MeDIP data viewing and access

MeDIP data were viewed using the Integrated Genomics Viewer (IGV) and deposited under NCBI GEO GSE66796.

### Chromatin immunoprecipitation (ChIP)

DNA and protein were cross-linked with 1% formaldehyde, cells lysed, chromatin fragmented by sonication under cooling for 4 × 8 min (30 sec ON/OFF) on “High” in a Bioruptor^™^ (Diagenode) to obtain chromatin fragments of 200–500 bp. After sedimentation, chromatin fractions from four donors were pooled. Dynabeads^®^ Protein A (Life Technologies) in RIPA buffer were mixed with 2.5 μg antibody (Diagenode: H3K4me3 pAb-003–050, H3K27me3 pAb-069–050, H3K27ac pAb-174–050 or H3K4me1 pAb-037–050) and incubated overnight at 4°C. Chromatin was diluted 1:4 in RIPA containing 1X protease inhibitor cocktail, 1 mM PMSF and 20 mM sodium butyrate. Approximately 35 A_260_ units of chromatin in 100 μl was used per ChIP and incubated with antibody-bead complexes for 2 h at 4°C. Immunoprecipitated material was RNase-treated with 33 ng/μl RNase (Roche) in elution buffer, digested with Proteinase K and isolated by phenol-chloroform-isoamylalcohol extraction. ChIP DNA was dissolved in 10 μl Milli-Q water. All ChIP DNA and 1/10 of input DNA was amplified by 25 cycles using the WGA4 kit (Sigma-Aldrich). Amplified ChIP DNA was purified with the QIAquick PCR Purification Kit (Qiagen) and eluted with 30 μl elution buffer (1/10 dilution in Milli-Q water). In order to analyze many promoters by quantitative (q)PCR, amplified ChIP material (2 μl of 1/100 dilution) was re-amplified and purified as above. We show that the reamplification of ChIP DNA before qPCR maintains the enrichment profiles of histone modifications tested (and RNA Pol II) on the models genes examined, i.e. MYOG and GAPDH (Supplementary Figure S5). Re-amplified ChIP DNA was analyzed by qPCR using IQ SYBR^®^ Green (BioRad) with ChIP primers listed in Supplementary Table S8. PCR conditions were 95°C for 3 min and 40 cycles of 95°C for 30 sec, 60°C for 30 sec and 72°C for 30 sec.

### Statistical analysis

GraphPad Prism v4.0.0 (GraphPad Software, La Jolla, CA) and GeneSpring GX12 (Agilent) were used for statistical analysis. Differences among groups were tested for statistical significance by One-way ANOVA with posthoc Tukey analysis or Student’s *t*-test. Differences were considered statistically significant when *P* values were < 0.05.

## SUPPLEMENTARY MATERIAL FIGURES AND TABLES















## Abbreviations

HEP: Hepatocyte
HSC: Hepatic stellate cell
qHSC: quiescent HSC
aHSC: activated HSC
LSEC: Liver sinusoidal endothelial cell
KC: Kupffer cell
ECM: Extracellular matrix
GO: Gene ontology
ChIP: Chromatin immunoprecipitation
KS: Kolmogorov-Smirnov
TSS: Transcription start site
CCl_4_: Carbon tetrachloride
H3K4me1: Histone 3 lysine 4 mono-methylation
H3K4me3: H3K4 tri-methylation
H3K27me3: Histone 3 lysine 27 tri-methylation
H3K27ac: H3K27 acetylation
DNMT1: DNA (cytosine-5)-methyltransferase 1
MeCP2: Methyl CpG binding protein 2
CpG: cytosine-phosphate-guanine
UV: Ultraviolet.
