# Supplementary material for: Genome-wide analysis of DNA methylation and gene expression patterns in purified, uncultured human liver cells and activated hepatic stellate cells
Source: Oncotarget. 2015 Aug 28;6(29):26729–45. doi: 10.18632/oncotarget.4925 (PMC4694948; doi:10.18632/oncotarget.4925)
Supplement: Supplementary file 1 [file oncotarget-06-26729-s001.pdf]

SUPPLEMENTARY FIGURES AND TABLES

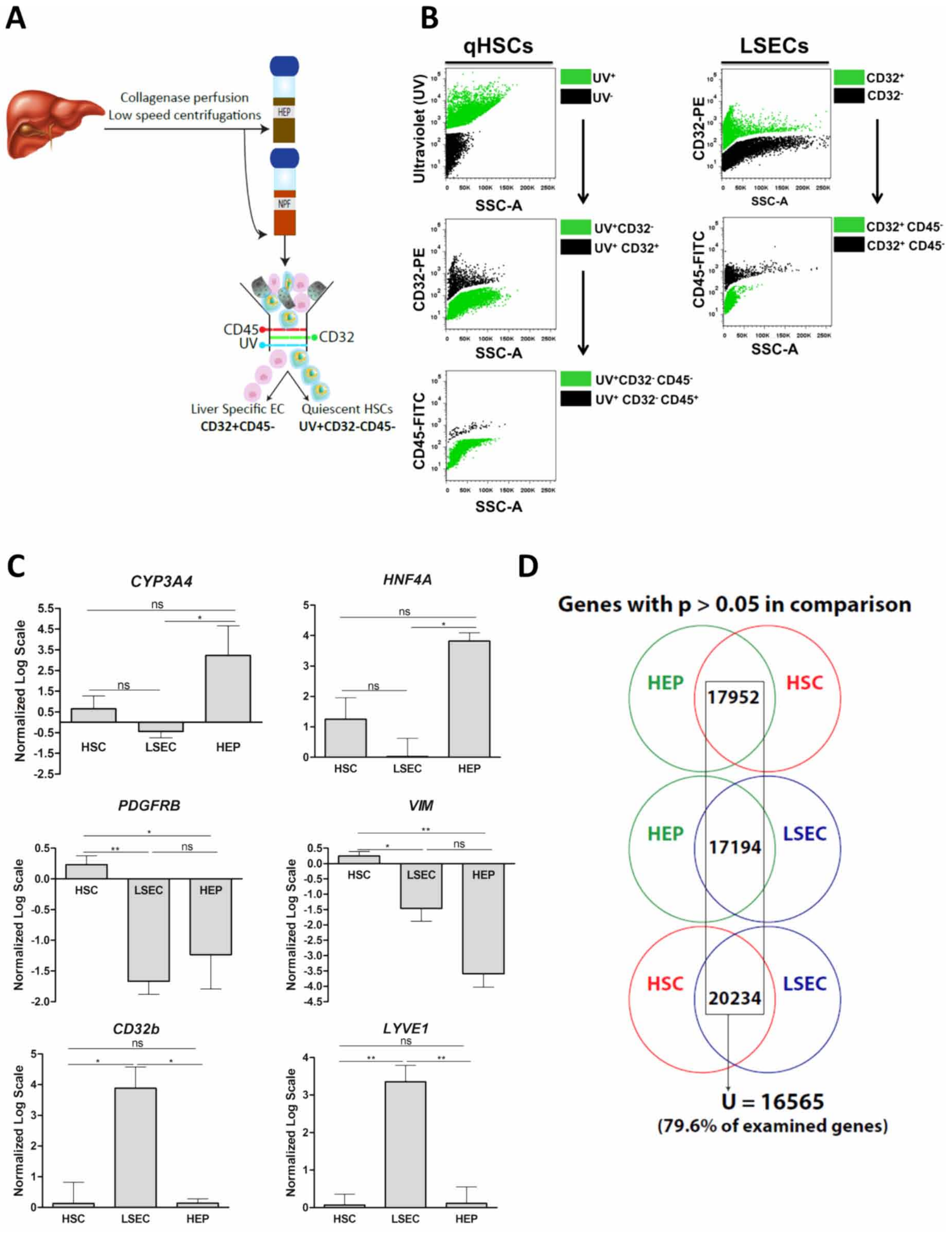

Supplementary Figure S1: High purity isolation of distinct human liver cell populations by combining collagenase digestion and flow cytometry. A. Schematic representation of the procedure that allows for the simultaneous purification of HEPs, HSCs and LSECs from human liver tissue. **B.** FACS profiles show the presence of CD32 and CD45 expressing cells in the high ultraviolet (UV) cell fraction. HSCs and LSECs were isolated as UV+CD32-CD45- cells and CD32+CD45- cells, respectively. **C.** Graphic representation of the expression level of genes known to be expressed in HEPs (*CYP3A4*, *HNF4A*), HSCs (*PDGFRB*, *VIM*) and LSECs (*CD32B*, *LYVE1*). Data is shown as normalized intensities, in a logarithmic scale. ns $P \geq 0.05$, $*P < 0.05$, $**P < 0.01$. **D.** Total number of genes with similar ($P > 0.05$, ANOVA) expression levels in each comparison. The union of these different set of genes represents ~80% of total genes examined ($n = 16565/20816$).

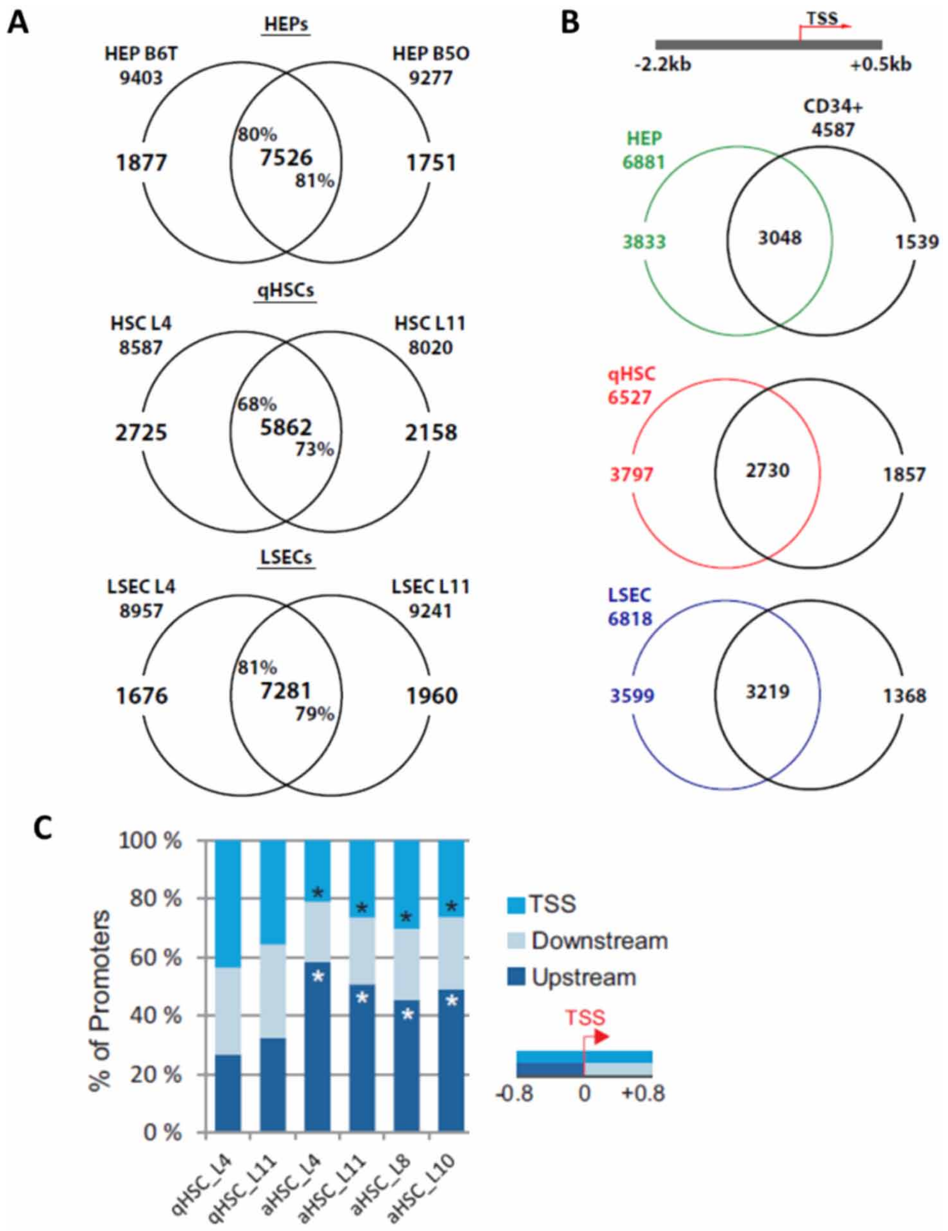

Supplementary Figure S2: A. DNA methylome comparison per donor of each cell type. **B.** Venn-diagram analysis of promoter methylation in CD34+ progenitors from bone marrow and HEPs, LSECs and HSCs. Analysis was focused on one donor for each liver cell type. **C.** Proportions of promoters showing DNA methylation upstream of the TSS, (−0.8 to TSS), downstream of the TSS (TSS to +0.8 kb), and over the TSS (both upstream and downstream). *$P \leq 0.0001$, Chi-square test with Yate's correction.

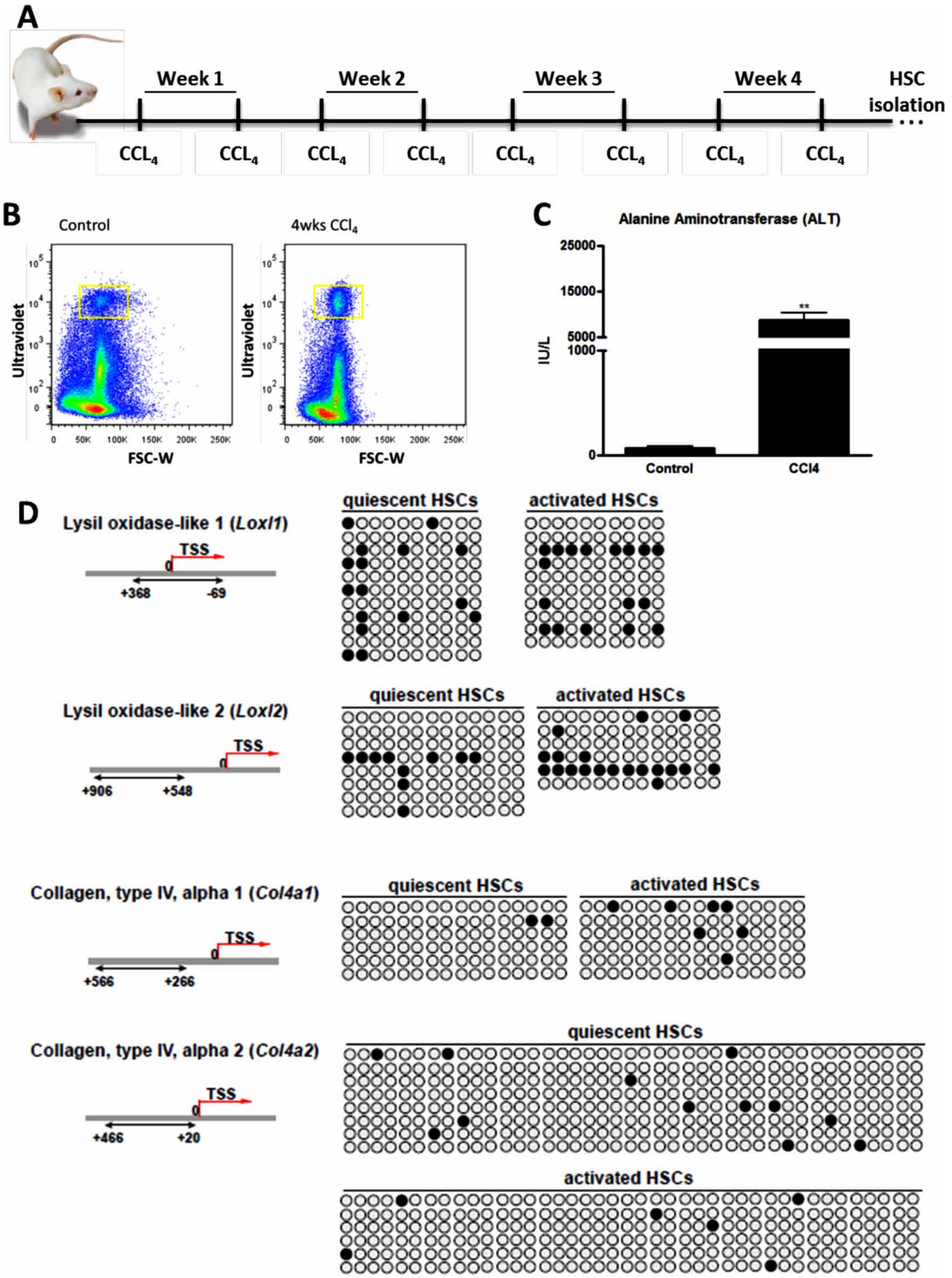

Supplementary Figure S3: A. For the *in vivo* activation of HSCs, healthy BalbC mice were injected with CCl_4 intraperitoneally twice a week for 4 weeks. Mice were sacrified for HSC isolation 24hrs after the last injection. Untreated mice of the same age were used as controls. **B.** HSCs were purified from healthy and fibrotic livers as high ultraviolet-positive cells. **C.** Serum alanine aminotransferase (ALT) levels measured from healthy and fibrotic mice. **D.** Bisulfite sequencing analysis of CpG methylation of the *Loxl1*, *Loxl2*, *Col4a1* and *Col4a2* promoters of HSCs purified from healthy and fibrotic mice.

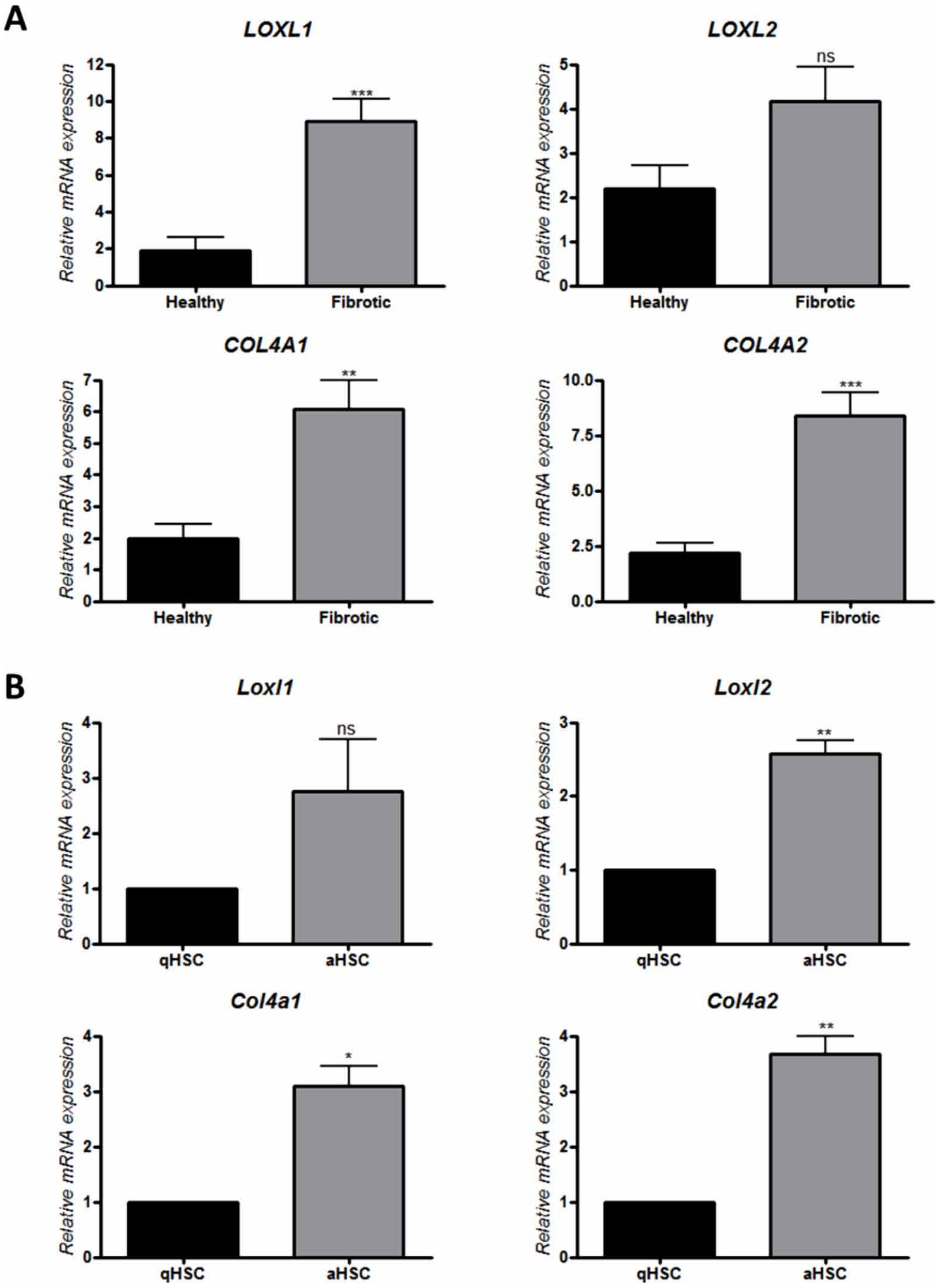

Supplementary Figure S4: A. Relative mRNA expression levels of *LOXL1*, *LOXL2*, *COL4A1* and *COL4A2* in total liver samples from healthy and fibrotic patients. **B.** Relative mRNA expression of *LoxL1*, *Loxl2*, *Col4a1* and *Col4a2* in mouse HSCs isolated from healthy and 4 weeks CCl_4-treated mice. *$P < 0.05$, **$P < 0.01$, ***$P < 0.001$.

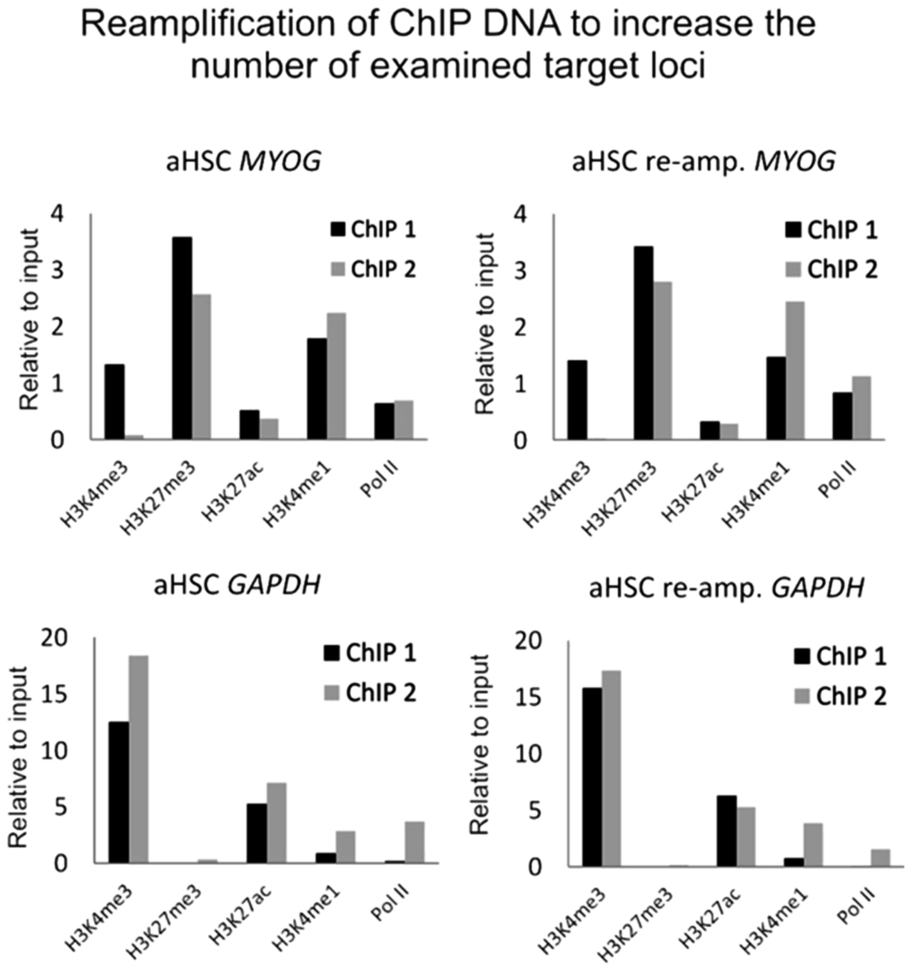

Supplementary Figure S5: Reamplification of ChIP DNA does not significantly affect the end qPCR result for the genes tested. Data show ChIP-qPCR data for histone modification and RNA Pol II enrichment levels on the MYOG (top) and GAPDH (bottom) promoters, for two independent ChIPs (1 and 2). Note the similarity of the profiles after one round (left panels) or two rounds (right panels) of amplification.

Supplementary Table S1: Genes specifically expressed in HEPs, HSCs and LSECs

Supplementary Table S2: GO terms of genes specifically expressed in HEPs, HSCs and LSECs

GO ACCESSION	GO Terms HEP	*p*-value	Count in Selection
GO:0044710	single-organism metabolic process	< 1E-44	379
GO:0044281	small molecule metabolic process	< 1E-44	234
GO:0055114	oxidation-reduction process	< 1E-44	154
GO:0006082	organic acid metabolic process	< 1E-44	142
GO:0043436	oxoacid metabolic process	< 1E-44	140
GO:0019752	carboxylic acid metabolic process	< 1E-44	138
GO:0006629	lipid metabolic process	< 1E-44	159
GO:0016491	oxidoreductase activity	6.7E-44	136
GO:0005739	mitochondrion	9.2E-44	198
GO:0003824	catalytic activity	3.9E-38	455
GO:0044255	cellular lipid metabolic process	3.7E-32	106
GO:0048037	cofactor binding	9.6E-32	71
GO:0044444	cytoplasmic part	1.2E-30	426
GO:0009055|GO:0009053|GO:0009054	electron carrier activity	2.3E-29	58
GO:0008202	steroid metabolic process	6.8E-29	62

GO ACCESSION	GO Term LSEC	*p*-value	Count in Selection
GO:0030246GO:0005529	carbohydrate binding	4.9E-5	7
GO:0006897|GO:0016193|GO:0016196	endocytosis	1.1E-4	7
GO:0030119	AP-type membrane coat adaptor complex	3.0E-4	3
GO:0030131	clathrin adaptor complex	2.8E-4	3
GO:0030118GO:0016190	clathrin coat	5.5E-4	3
GO:0008565|GO:0015463	protein transporter activity	6.9E-4	4
GO:0000904	cell morphogenesis involved in differentiation	0.6	1
GO:0000910|GO:0007104|GO:0016288|GO:0033205	cytokinesis	0.2	1
GO:0001501	skeletal system development	0.7	1
GO:0001525	angiogenesis	0.5	1
GO:0001568	blood vessel development	0.7	1
GO:0001605	adrenomedullin receptor activity	0.004	1
GO:0001608	G-protein coupled nucleotide receptor activity	0.1	1
GO:0001614	purinergic nucleotide receptor activity	0.1	1
GO:0001637	G-protein coupled chemoattractant receptor activity	0.1	1

(*Continued*)

GO ACCESSION	GO Term HSC	*p*-value	Count in Selection
GO:0044421	extracellular region part	2.1E-11	19
GO:0031012	extracellular matrix	4.5E-10	12
GO:0005578	proteinaceous extracellular matrix	2.1E-9	11
GO:0005576	extracellular region	2.8E-8	22
GO:0048731	system development	2.0E-7	23
GO:0030185	nitric oxide transport	2.2E-7	3
GO:0048513	organ development	2.1E-7	19
GO:0005201	extracellular matrix structural constituent	1.6E-7	6
GO:0032502	developmental process	2.1E-7	27
GO:0048856	anatomical structure development	1.5E-7	25
GO:0001501	skeletal system development	2.6E-7	9
GO:0045766	positive regulation of angiogenesis	3.7E-7	5
GO:0007275	multicellular organismal development	5.3E-7	25
GO:0044767	single-organism developmental process	6.3E-7	26
GO:0032501|GO:0050874	multicellular organismal process	1.1E-6	30

Supplementary Table S3: Methylated genes in HEPs, HSCs and LSECs

Supplementary Table S4: GO terms of genes specifically methylated in HEPs, HSCs and LSECs

Supplementary Table S5: Genes specifically methylated in HEPs, HSCs and LSECs

Supplementary Table S6: Genes differentially expressed in human qHSCs and aHSCs

Supplementary Table S7: Genes with concordant changes in DNA methylation and gene expression upon HSC activation

Supplementary Table S8: ChIP and bisulfite methylation PCR primers used in this study

Gene	Forward primer (F) 5′→3′<br>Reverse primer (R) 5′→3′	Position rel. to TSS (nt)	Annealing temp. (°C)
**ChIP-PCR primersv**			
ACTA2	F: TCCCGTTTCATGAGCAGACC<br>R: CTCTCTAATCTGGGTGGCCG	−206/−135	60
ACTG2 (HG18)	F: ACAGCTGCATCTGGTCTCAC<br>R: CCCGGTGCTGGTAGATTTGT	−265/−166	60
ADAMTS12	F: GGAAGAGAATCCCAGAGCGG<br>R: CCCTGCTGTTCTCCTCCAAG	−347/−256	60
APOB	F: ACTGAATTGGGAGCCACGAG<br>R: GGATTAGGGTGTGGGTGCAA	−471/−352	60
COL1A1	F: TTAGCCCACGCCATTCTGAG<br>R: ACCCCACATCTCCCCTCTTC	−281/−171	60
COL1A2	F: CCACGCTATCGAGTCTTCCC<br>R: GCCCTGTAGGCCACTTGTAG	−171/−87	60
COL3A1	F: GCATACAAACTCCAGATGTGCTC<br>R: GAAATATGAGAGCCGCACCC	−133/−16	60
COL4A1	F: CAAAGCGAGTTTAGCGCAGG<br>R: TCTCCTTCTTTCCGGGTCGT	−696/−552	60
COL4A1 Intragenic Enhancer 1	F: AGGCGCTAAATGGCTGGTAA<br>R: GGAGAAGTCTGTGGCCAGTC	~ + 69 kb	60
COL4A1 Intragenic Enhancer 2	F: GGTCCAAAAATCCATGGCGG<br>R: GGTTCAGCTGCACGAAAACT	~ + 90 kb	60
LOX	F: CCTTAACGCTCCCTGTGCAA<br>R: CGATTGGAAACGTGCAAGGC	−178/−76	60
LOXL1	F: GAGAAGCCAGTGAGCAAGGT<br>R: GAGTAGGAGGGGACCTCTGG	−330/−185	60
LOXL1 IntragenicEnhancer	F: GAGAGGCCAGGAACAGCATT<br>R: GCTATGCAACCCAGGGAAGT	~ + 14 kb	60
LOXL1 Downstream Enhancer	F: TCATGGCTTACAGTGCCTCG<br>R: GCTGGAACACAGTAAGGGCT	~ +39 kb	60
LOXL2	F: GGCCATAGACGTGATCTGCT<br>R: ACGCTCAATTAAAGCTGCCG	−413/−268	60
LOXL2 Upstream Enhancer	F: AGACCAGACCCAGACCAAGT<br>R: GCAGATGCGTCCTAACCACT	~ − 6,5 kb	60
NOTCH1	F: GGCTCCTCCGCTTATTCACA<br>R: GCCAAAAGTTTGAGCCGGG	528/−413	60
TGFB1	F: TCCTTCAGGTGTCCTGTTGC<br>R: GAGGGTCTGTCAACATGGGG	−483/−413	60

(*Continued*)

Gene	Forward primer (F) 5′→3′<br>Reverse primer (R) 5′→3′	Position rel. to TSS (nt)	Annealing temp. (°C)
**Human bisulfite PCR primers**			
ACTG2	F: TATTTATTTTGGGGATAGTGTTTGG<br>R: TCACACCAAACCTTATAAACTACCC	−394/−217	58
APOB	F: GGTATAATTGTTGGGGGATTATTTAG<br>R: CTTCTAAAATCATCAATCTTCACCAA	−1319/−1121	58
**Mouse bisulfite PCR primers**			
Col4a1	F: GTAAAGGTGGTAGGTAGAGAGAGTAT<br>R: AACAACAACTCCTAACCCTAAAATAAA	−566/−266	58
Col4a2	F: GTTGTTTATAGGGATTAGGAGAGGG<br>R: CCAACAAATTAAAAAACTCCTC	−466/−20	58
Loxl1	F: GGAGAAGGGAGAAGGTATTTTAAGA<br>R: CATAACCAACCCCACACTAACTAAC	−368/+69	58
Loxl2	F: TTGTTTGAGAAGTTTTTGGGTAGAT<br>R: AATTCTACACTCTCCACCTCCTAAC	−906/−548	58
